# Dexmedetomidine alleviates inflammatory response and oxidative stress injury of vascular smooth muscle cell via α2AR/GSK-3β/MKP-1/NRF2 axis in intracranial aneurysm

**DOI:** 10.1186/s40360-022-00607-0

**Published:** 2022-10-23

**Authors:** Ze Zhang, Xiue Mu, Xiaohui Zhou

**Affiliations:** Department of Anesthesiology, The First Hospital of Hebei Medical University, 89 Donggang Road, Shijiazhuang, 050000 Hebei China

**Keywords:** Intracranial aneurysm, Dexmedetomidine, Inflammation, Oxidative stress, Vascular smooth muscle cells

## Abstract

Vascular smooth muscle cell (VSMC) phenotypic modulation regulates the initiation and progression of intracranial aneurysm (IA). Dexmedetomidine (DEX) is suggested to play neuroprotective roles in patients with craniocerebral injury. Therefore, we investigated the biological functions of DEX and its mechanisms against IA formation and progression in the current study. The rat primary VSMCs were isolated from Sprague–Dawley rats. IA and superficial temporal artery (STA) tissue samples were obtained from patients with IA. Flow cytometry was conducted to identify the characteristics of isolated VSMCs. Hydrogen peroxide (H_2_O_2_) was used to mimic IA-like conditions in vitro. Cell viability was detected using CCK-8 assays. Wound healing and Transwell assays were performed to detect cell motility. ROS production was determined by immunofluorescence using DCFH-DA probes. Western blotting and RT-qPCR were carried out to measure gene expression levels. Inflammation responses were determined by measuring inflammatory cytokines. Immunohistochemistry staining was conducted to measure α_2_-adrenergic receptor levels in tissue samples. DEX alleviated the H_2_O_2_-induced cytotoxicity, attenuated the promoting effects of H_2_O_2_ on cell malignancy, and protected VSMCs against H_2_O_2_-induced oxidative damage and inflammation response. DEX regulated the GSK-3β/MKP-1/NRF2 pathway via the α2AR. DEX alleviates the inflammatory responses and oxidative damage of VSMCs by regulating the GSK-3β/MKP-1/NRF2 pathway via the α2AR in IA.

**Supplementary Information:**

The online version contains supplementary material available at 10.1186/s40360-022-00607-0.

## Introduction

Intracranial aneurysm (IA) is a regional bulging on intracranial artery characterized by asymptomatic lesion [1]. Rupture of IA may lead to subarachnoid hemorrhage (SAH) accompanied by severe headache, vomiting and consciousness impairment [2, 3]. The mortality rate of IA-induced SAH is 50% worldwide. The living quality of only 30%-45% survivors returns to their pre-onset state [4]. History of hypertension, genetics, sex, age, and cigarette smoking are potential contributors of aneurysm rupture [5]. The abnormal hemodynamic changes are considered as the initiating factor of endothelial cell dysfunction [6]. Despite this contributor, inflammatory responses, oxidative damage and cell death are associated with IA etiology [7]. The stimulated inflammatory mediators release numerous inflammatory cytokines and oxidative factors, resulting in the phenotype modulation of vascular smooth muscle cell (VSMC) and remodeling of dysfunctional extracellular matrix (ECM) [8]. Microsurgery and endovascular interventions have made significant advances in treating IA, but morbidity and mortality remain formidably high. Therefore, developing noninvasive pharmacological therapies to prevent the progression and rupture of IA is of great significance.

VSMCs, a major cell type in vessel walls, is well-known for their multifunctional properties, including maintaining the vessel integrity, regulating blood pressure, and redistributing blood flow [9, 10]. VSMCs involve two types: contractile and synthetic. Smooth muscle 22 alpha (SM22 α), smooth muscle alpha actin (αSMA), SM myosin heavy chain (MHC), h1-calponin and smoothelin are markers of contractile phenotype [11]. The inflammation and oxidative stress cause the conversion from contractile phenotype into synthetic type [12]. Under normal physiological environment, there is a dynamic balance between the proliferation and apoptosis of VSMCs. Endothelial dysfunction and phenotypic transformation of VSMCs contribute to the initiation and development of IA [13].

Dexmedetomidine (DEX), a highly selective α_2_-adrenergic receptor (α2AR) agonist, exhibits sympatholytic, sedative, amnestic and analgesic properties [14, 15]. DEX can serve as an auxiliary analgesic to strengthen the sedative and analgesic effects, can maintain hemodynamic stability, and can reduce the necessary dosage of other analgesic agents [16, 17]. A previous study has elucidated that during the operation of craniocerebral patients, DEX can ameliorate the surgery-induced harmful stimulation and improve cerebral blood flow [18]. In patients with craniocerebral injury, DEX also inhibits the generation of inflammatory mediators, improves cerebral oxygen, and alleviates oxidative damage, thereby effectively enhancing the vessel stability and reducing the craniocerebral injury-induced brain edema [19]. DEX plays antioxidant and anti-apoptotic roles in cerebral ischemia and hypoxia to alleviate brain damages and restore the function of perioperative brain in patients with IA [20]. In an in vivo model of SAH, the DEX-mediated reduction in blood–brain barrier permeability leads to a better neurological outcome [21]. Furthermore, as reported, in patients with SAH, the clinical application of DEX is promising in neurocritical care and diagnostic cerebral angiography [22, 23]. However, the biological functions of DEX and its related mechanisms in IA progression have not been fully understood.

Multiple proteins participate in the regulation of the antioxidant response, including glycogen synthase kinase 3β (GSK-3β), mitogen-activated protein kinase phosphatase-1 (MKP-1), and the nuclear factor erythroid 2-related factor 2 (NRF2) [24, 25]. GSK-3β, ubiquitously expressed in eukaryotes, possesses multiple functions in various cellular behaviors, such as cell proliferation, differentiation, and apoptosis [26]. GSK-3β is inactivated by phosphorylation at Serine 9 residue (Ser9) and activated by phosphorylation at Tyrosine 216 residue (Tyr216) [27]. Activated MAPK promotes the proliferation of VSMCs, and MKP-1 is a critical contributor of the inactivated MAPK pathway [28, 29]. NRF2 activation promotes the production of the antioxidant response element (ARE), inducing the reduction in the oxidative stress [30].

Hydrogen peroxide (H_2_O_2_), an inducer of oxidative stress, is involved in apoptosis of VSMCs [31]. H_2_O_2_ has been used to establish an in vitro model of IA via inducing the apoptosis of VSMCs [32–34]. Therefore, we herein investigated the biological functions of DEX and its related mechanisms in a H_2_O_2_-induced cellular model of IA. We hypothesized that DEX might prevent progression of rupture of IA. We believed that our study would provide theoretical reference for IA treatment.

## Methods

### Patients and tissue samples

Before the study, written informed consents were obtained from the patients, and this study was granted approval of the Ethics Committee of The First Hospital of Hebei Medical University (Ethical approval: 20,190,506). All participates were over 16 years of age. The study protocols were also based on the ethical principles of the Declaration of Helsinki for medical research involving human subjects. 80 patients with IA participated in this study. IA tissue samples were obtained during the surgery, and superficial temporal artery (STA) vascular tissue samples were collected as controls. All samples were fixed in 10% formaldehyde (Yuanmu Biotech, Shanghai, China) and embedded in paraffin.

### VSMC isolation and cell culture

Sprague–Dawley rats (male, 8 weeks old, 300–320 g) were purchased from Hunan SJA Laboratory Animal CO., LTD (Hunan, China). All experimental procedures were based on the National Institutes of Health Guidelines for the Care and Use of Laboratory Animals and approved by the Ethics Committee of the First Hospital of Hebei Medical University (Ethical approval: 2019-DWLL0506A). All methods were conducted in accordance with the ARRIVE guidelines (https://arriveguidelines.org). Rat brain VSMCs were separated from the Circle of Willis. The brain vessels were collected under sterile conditions followed by phosphate buffer solution (PBS; Absin Biotechnology, Shanghai, China) washing. After washing, excess fat and fascia were removed. Subsequently, the vessels were placed in enzyme solution containing 0.2% collagenase II and 1 mg/ml soybean trypsin inhibitor for 20 min at 37℃. For VSMCs isolation, the blood vessels were cut off along the long axis and the intima was tore off. Next, the vessels were cut into pieces (1 mm^3^) and 0.125% trypsin was added. After incubation for 15 min at 37℃, DMEM (Sunncell Biotech, Hubei, Wuhan, China) supplemented with 10% fetal bovine serum (FBS; Excell Biotechnology, Shanghai, China) was added. Then the digested VSMCs were collected by centrifugation and the supernatant was removed. The collected cells were cultured in DMEM supplemented with 10% FBS, 100U/ml penicillin, and 100 mg/ml streptomycin at 37℃ with 5% CO_2_ in a humidified condition. After 3 days of primary culture, the cells grew adhering to the wall. After 2 weeks, the cells were observed to be spindle shaped using a light microscope (Imager D2, ZEISS, Germany). VSMCs at passages 3 to 8 were used for subsequent experiments.

### VSMC identification

The SMC differentiation markers and stem cell markers using flow cytometry. The cells were cultured in a Fluorescence-Activated Cell Sorting (FACS) buffer containing 2% FBS and PBS. After fixation and permeabilization, SMC-specific markers myosin heavy chain 11 (Myh11; ab224805, 1:80; Abcam), calponin 1 (ab46794, 1:100; Abcam), Smoothelin2 (ab236034, 1:50; Abcam) and stem cell markers CD44 (ab238464, 0.474 μg/ml; Abcam), stem cell antigen-1 (Sca1; ab51317, 0.2 μg/10^6^ cells; Abcam) and S10 calcium-binding protein B (S100β; Thermo Fisher Scientific, Waltham, MA, USA) were added. All antibodies were used at the manufacturer’s recommended dilutions and cellular concentrations. Subsequently, the signals of labelled cells were detected by flow cytometry and the data were analyzed by FlowJo software (Beckman Coulter, Fullerton, CA, USA).

### Cell treatment and CCK-8 assays

The first step of the study was to find out the optimal concentration of hydrogen peroxide (H_2_O_2_) and DEX by conducting CCK-8 assays. The VSMCs were first exposed to different concentrations (0, 0.1, 1, 10 μM) of DEX for 120 min. The treated cells seeded in the 96-well plates (5 × 10^3^ cells/well) until 80% confluence. Then 10 μl of CCK-8 reagent (Yeasen Biotechnology, Shanghai, China) was added into the culture medium. Another 2 h was given the cells for incubation at 37℃. Absorbance at 450 nm was measured using a microscope (Bio-Tek, Winooski, VT, USA). To identify optimal concentration of H_2_O_2_, the cells were exposed to different concentrations (0.2, 0.5, 1, 10 mM/ml) of H_2_O_2_ for 12 h, and then CCK-8 assays were conducted as described above. To identify optimal concentration of DEX, we pretreated VSMC with different concentrations (0, 0.1, 1, and 10 μM) DEX for 2 h before exposure to 1 mM/ml H2O2 for 12 h. Then 10 μl of CCK-8 reagent was added, and the optimal concentration of DEX was obtained based on cell viability. The optimal concentration of DEX and H_2_O_2_ was 1 μM and 1 mM/ml, respectively. Then the VSMCs were divided into the control group (DMEM), the DEX group (1 μM DEX), H_2_O_2_ group (1 mM/ml H_2_O_2_), H_2_O_2_ + NS group (1 mM/ml H_2_O_2_ + normal saline), H_2_O_2_ + DEX group (1 mM/ml H_2_O_2_ + 1 μM DEX), the H_2_O_2_ + DEX + BRL (α2AR inhibitor, group and the H_2_O_2_ + SB (GSK-3β inhibitor) group.

### Intracellular ROS measurement

The VSMCs cells were seeded into 6-well plates (1 × 10^5^ cells/well) to measure the intracellular ROS levels. After treatment as described above, PBS washing was conducted. The cells were then incubated at 37℃ with 10 μM DCFH-DA (Beyotime, Shanghai, China) for 1 h. Next, the VSMCs were washed three times using the serum-free DMEM and suspended in PBS. A fluorescence microscope (Leica Biosystems, Shanghai, China) was used to visualize the fluorescence intensity of DCF at 488 nm (excitation) and 525 nm (emission).

### Wound healing assays

The cells were seeded into 24-well plates (5 × 10^4^ cells/well) and incubated at 37℃ with 5% CO_2_ for 24 h. Until the cells reached 90% confluence, a pipette tip (10 μl) was used to make scratches on the cells. The cells were then washed using PBS and subsequently cultured at 37℃ with 5% CO_2_ for 24 h. The wound width was imaged using an AxioCam camera (Carl Meditec, Jena, Germany) at 0 and 24 h. ImageJ software was used to evaluate the remaining area [35].

### Transwell assays

The cells were plated in transwell inserts with 8-μM pore size in serum free DMEM. DMEM with 10% PBS was added to the lower chamber. After 12 h, the cells on the upper side of the insert were removed and the migrated cells were fixed with 4% paraformaldehyde (Sigma-Aldrich) and stained with crystal violet. Number of migrated cells were measured using an inverted microscope (Leica). Six fields were randomly selected, and the average was calculated [36].

### Western blotting

The proteins were collected using RIPA lysis buffer (Sigma-Aldrich) containing protease inhibitor. Cell lysates were resolved by sodium dodecyl sulfate–polyacrylamide gel electrophoresis and then transferred onto polyvinylidene fluoride membranes (PVDF). After blocking with 5% skimmed milk, the membranes were incubated overnight with primary antibodies against SM22α (ab14106, 1 μg/ml; Abcam), αSMA (ab7817, 0.341 μg/ml; Abcam), α2AR (ab85570, 1 μg/ml; Abcam), p-GSK-3β (ab107166, 1 μg/ml; Abcam), GSK-3β (ab93926, 1:1000; Abcam), MKP-1 (ab138265, 1:1000; Abcam), NRF2 (ab92946, 1:1000; Abcam) and GAPDH (ab8245, 1:1000; Abcam) at 4℃. Then the membranes were washed three times. After that, the membranes were incubated with secondary antibodies for 2 h at room temperature. After being probed with enhanced chemiluminescence (Yeasen Biotechnology), the proteins were visualized using an image analysis system (Tanon, Shanghai, China). The protein intensity was evaluated by ImageJ software.

### RT-qPCR

IA and STA tissue samples were stored at -80℃ and placed in pre-cooled lysis buffer (Sigma-Aldrich) after stirring with an electric homogenate machine. After centrifugation at 1200 rpm for 30 min, the supernatant was collected. TRIzol (Sigma-Aldrich) was used for total RNA isolation from lysed tissues and VSMCs. Reverse transcription into cDNA was done using the Reverse Transcription Kit (Sigma-Aldrich). RT-qPCR was performed on the ABI 7300 system (Applied Biosystems, Foster City, UA, USA). The relative level was calculated using 2^−△△Ct^, and expressed as ratio of GAPDH. The primer sequences were listed in Table 1.

**Table 1:** Sequences of primers used for reverse transcription-quantitative PCR

Gene	Sequence (5′3’)
NQO-1 forward	AGGCTGGTTTGAGCGAGT
NQO-1 reverse	ATTGAATTCGGGCGTCTGCTG
GCLC forward	ACAGCACGTTGCTCATCTCT
GCLC reverse	TCATCCACCTGGCAACAGTC
SOD1 forward	GCGTCATTCACTTCGAGCAG
SOD1 reverse	TTCCACCTTTGCCCAAGTCAT
α2AR forward	GTCATCGGAGTGTTCGTGGT
α2AR reverse	GTGAGCCATGCCCTTGTAGT
GAPDH forward	GCAAGTTCAACGGCACAG
GAPDH reverse	GCCAGTAGACTCCACGACAT

### Measurement of TNF-α, IL-6 and MCP-1 levels

Cellular supernatants were collected after 1000 g centrifugation for 5 min. Interleukin-1β, IL-6 and tumor necrosis factor (TNF-α) ELISA kits (Enzyme-linked Biotechnology, Shanghai, China) were applied to measure the levels of inflammatory cytokines according to the kit instructions.

### Immunohistochemistry

The paraffin-embedded IA and STA tissue sections were cut into 5 μM and then placed on poly-L-lysine coated slides. For immunohistochemistry, the slides were incubated with primary antibody against α2AR (ab85570, 1 μg/ml; Abcam) overnight at 4℃. After washing, the slides were incubated with secondary antibody. The slides were visualized using a DAB plus chromogen (Thermo Fisher Scientific). Under 400 × magnification, the pictures were taken in 5 random fields.

### Statistics analysis

Each experiment was performed in triplicate. Statistical analyses were performed using the GraphPad Prism 6.0 software (GraphPad, Sam Diego, CA, USA). Data were described as the mean ± standard deviation. Student’s *t* test and one way analysis of variance followed by Tukey’s post hoc test were used to compare differences. *p* < 0.05 was considered statistically significant.

## Results

### Isolation and characterization of VSMCs

**Fig. 1:**
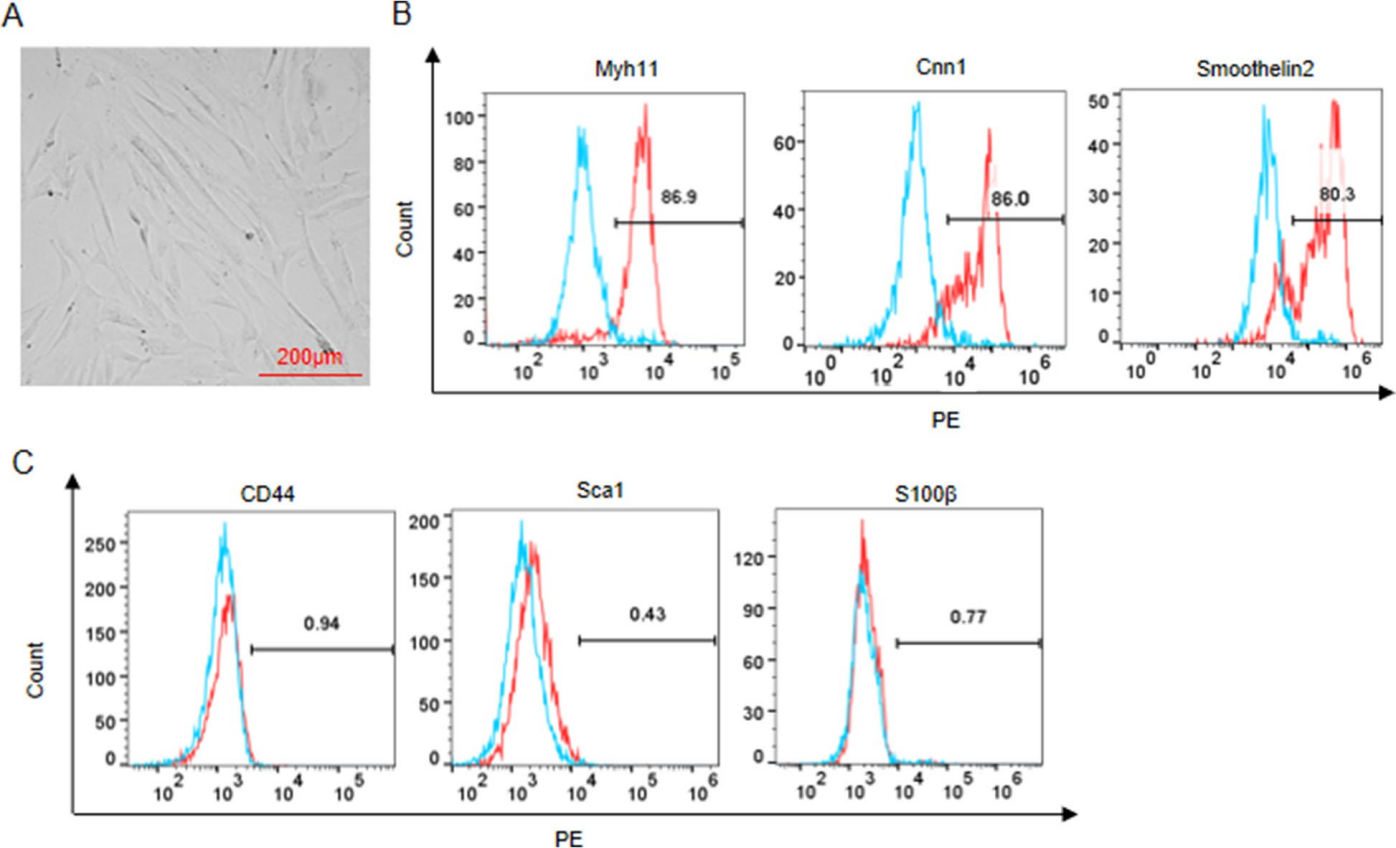
Isolation and characterization of VSMCs. **A** Morphology of VSMCs. **B** Flow cytometry of SMC-specific markers (Myh11, Cnn1, and Smoothelin2). **C** Flow cytometry of stem cell markers (CD44, Sca1, and S100β)

After 2 weeks, the cells were observed to be spindle shaped as shown in Fig. 1A. As flow cytometry revealed, the isolated VSMCs positively expressed SMC-specific markers, including Myh11, Cnn1, and Smoothelin2, while were negative for the stem cell markers, including CD44, Sca1, and S100β (Fig. 1B-C). These results demonstrate that the VSMCs were successfully isolated from blood vessels.

### DEX reverses the inhibitory effect of H_2_O_2_ on cell viability

**Fig. 2:**
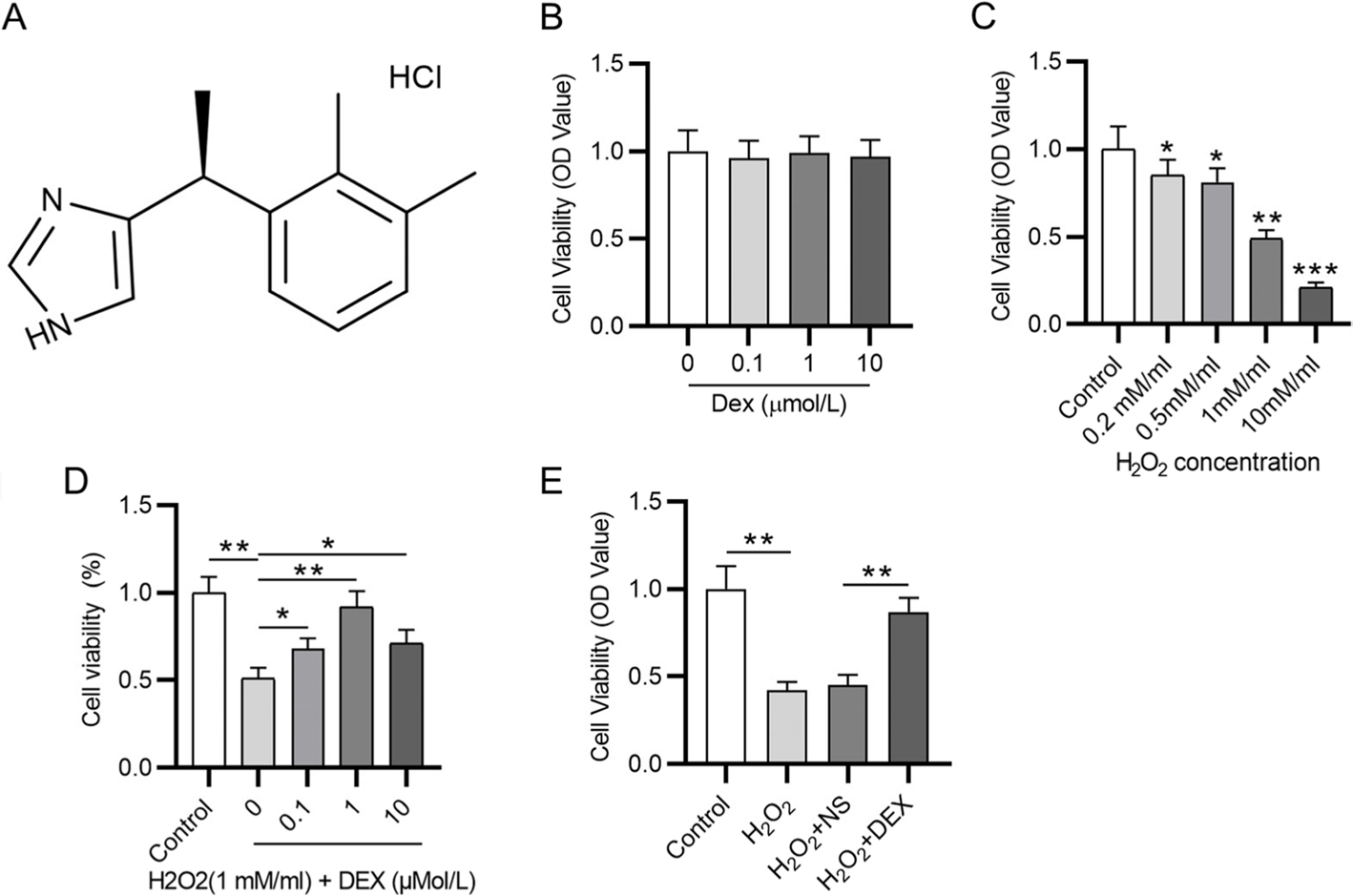
DEX alleviates the H_2_O_2_-induced reduction in cell viability. **A** Chemical structure of DEX. **B** The viability of VSMCs treated with different concentrations (0, 0.1, 1, 10 μM) of DEX was detected by CCK-8. **C** Viability changes of VSMCs after treatment with different concentrations (0.2, 0.5, 1, 10 mM/ml) of H_2_O_2_. **D** Viability of VSMCs after treatment with 1 mM/ml H_2_O_2_ and different concentrations of DEX (0, 0.1, 1, 10 μM). **E** Viability of VSMCs in the control group, the H_2_O_2_ group, H_2_O_2_ + NS group and the H_2_O_2_ + DEX group. *p < 0.05, **p < 0.01, ***p < 0.001. DEX, dexmedetomidine

Figure 2A presented the chemical structure of DEX. To detect DEX influence on cell viability, CCK-8 assays were carried out and the results demonstrated that compared with exposure to 0 μM DEX, no significant change of cell viability was observed when the VSMCs were exposed to 0.1–10 μM DEX (Fig. 2B). Then we detected the effects of H_2_O_2_ on the viability of VSMCs. Compared with that in the control group, the viability of VSMCs was reduced with increasing concentration of H_2_O_2_, as CCK-8 revealed (Fig. 2C). Afterwards, the cells were treated with 1 mM/ml H_2_O_2_ and different concentrations of DEX, and the CCK-8 results demonstrated that the protective effect of DEX against H_2_O_2_-induced cytotoxicity was optimal at the concentration of 1 μM (Fig. 2D). Finally, the results of CCK-8 showed that DEX rescued the reduction in cell viability caused by H_2_O_2_ (Fig. 2E). Above findings suggest that the H_2_O_2_-induced decrease in cell viability was reversed by DEX.

### DEX attenuates the promoting effects of H_2_O_2_ on cell malignancy

**Fig. 3:**
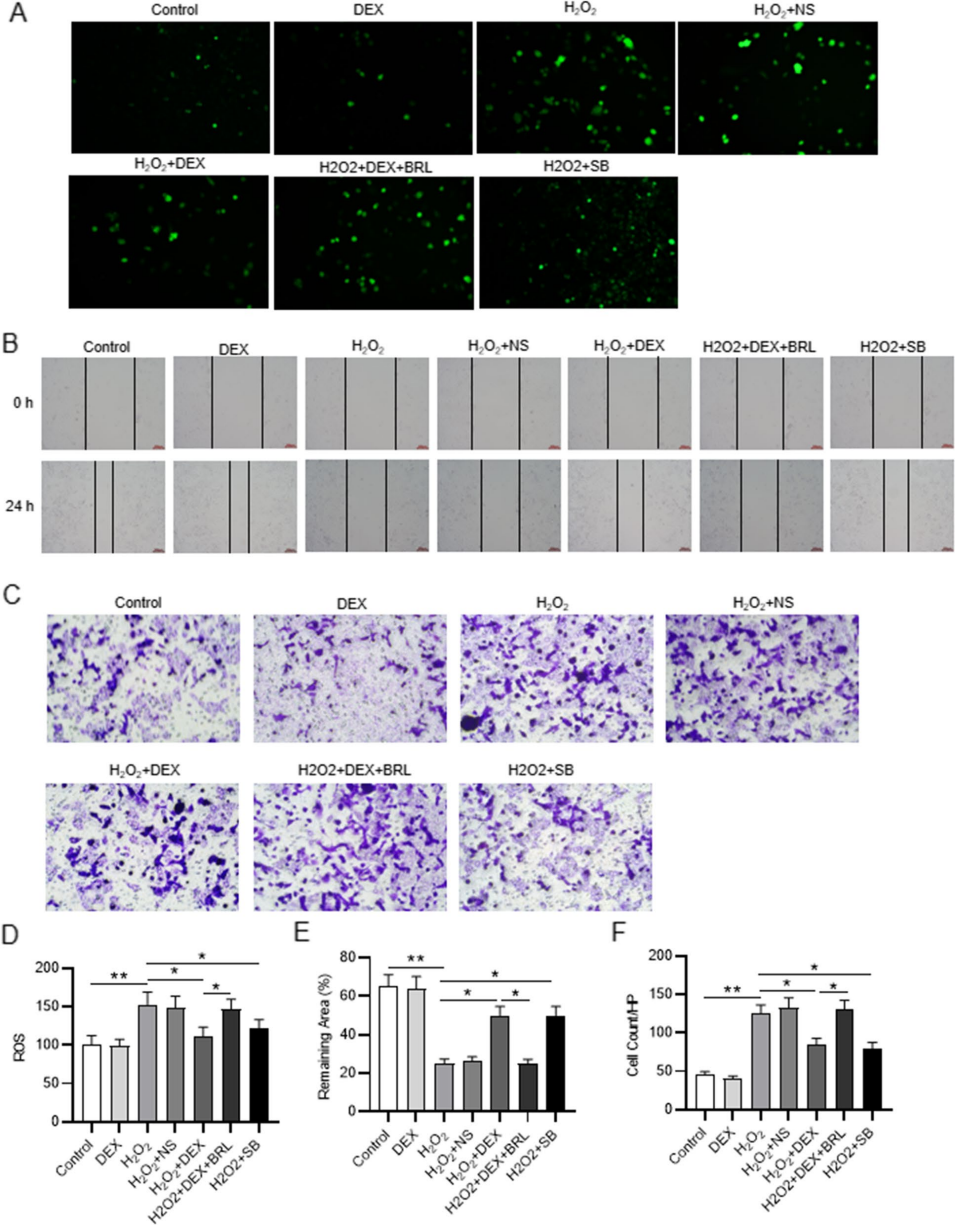
DEX attenuates the promoting effects of H_2_O_2_ on cell malignancy. **A** Immunofluorescence of intracellular ROS in the control group, the DEX group, the H_2_O_2_ group, H_2_O_2_ + NS group, the H_2_O_2_ + DEX group, the H_2_O_2_ + DEX + BRL group, and the H_2_O_2_ + SB group. **B** Wound healing assays of cell migration in indicated groups. **C** Transwell assays of cell invasion in different groups. *p < 0.05, **p < 0.01. DEX, dexmedetomidine; BRL, BRL44408, α2AR inhibitor; SB, SB216763, GSK-3β inhibitor. **D** Quantification of ROS production. **E** Quantification of the percentage of remaining area. **F** Quantification of the number of invaded cells

The intracellular ROS level was elevated following H_2_O_2_ treatment, while DEX or SB treatment restored the promotion in ROS production, as shown by the representative fluorescence images of ROS generation probed by DCFH-DA. Additionally, the α2AR inhibitor (BRL) reversed the inhibitory effects of DEX (Fig. 3A, D). DEX or SB treatment attenuated the inhibitory effects of H_2_O_2_ on the percentage of remaining area, indicating that DEX inhibits cell migration. However, BRL restored the DEX-induced promotion, as wound healing assays demonstrated (Fig. 3B, E). Afterwards, the increased invaded cells induced by H_2_O_2_ were decreased by DEX or SB treatment, and BRL further counteracted the suppressive effects of DEX, as transwell assays showed (Fig. 3C, F). These results suggest that DEX ameliorates the H_2_O_2_-induced malignant phenotypes of VSMCs via α2AR.

### DEX protects VSMCs against H_2_O_2_-induced oxidative damage and inflammation response

**Fig. 4:**
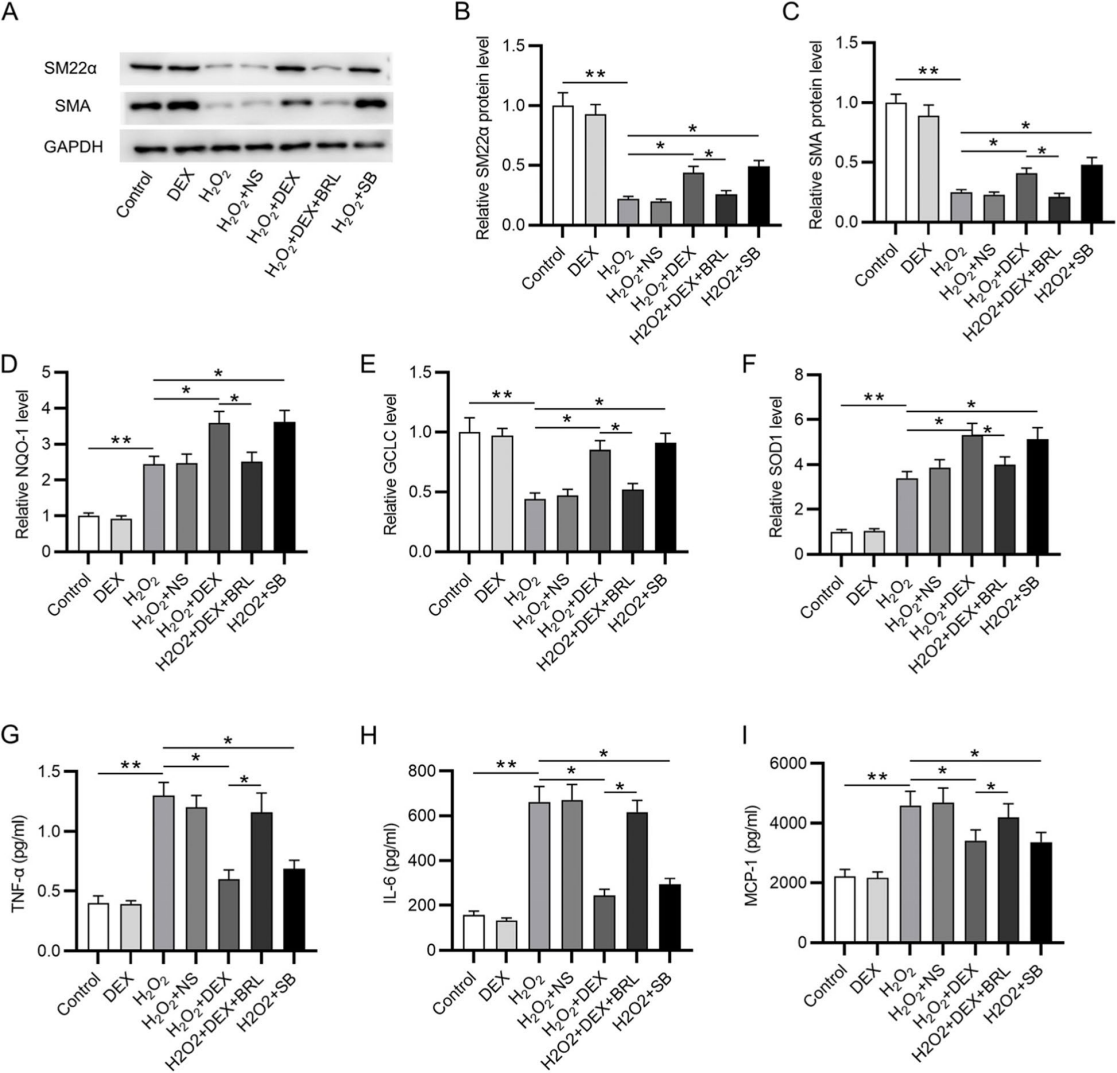
DEX protects VSMCs against H_2_O_2_-induced oxidative damage and inflammation response. **A**-**C** Western blotting was conducted to measure the protein levels of SM22α and αSMA. **D**-**F** Measurement of NQO-1, GCLC and SOD1 levels. **G**-**I** Quantification of TNF-α, IL-6 and MCP-1 levels. *p < 0.05, **p < 0.01. DEX, dexmedetomidine; BRL, BRL44408, α2AR inhibitor; SB, SB216763, GSK-3β inhibitor

Then we conducted western blotting to measure the levels of SM22α and αSMA (contractile phenotype markers). We found that H_2_O_2_ decreased the protein levels of contractile phenotype markers, while DEX or SB treatment attenuated the inhibitory effects of H_2_O_2_. However, BRL attenuated the promoting capability of DEX (Fig. 4A-C). As RT-qPCR revealed, DEX or SB restored the H_2_O_2_-induced promotion in the levels of NQO-1 and SOD1 and rescued the H_2_O_2_-induced reduction in the level of GCLC. Nevertheless, BRL played an opposite role against DEX (Fig. 4D-F). Finally, we found that DEX or SB treatment reversed the promoting effects of H_2_O_2_ on TNF-α, IL-6 and MCP-1 levels, which were then increased in response to BRL treatment (Fig. 4G-I). In summary, DEX alleviates the oxidative damage and inflammatory responses, suggesting that DEX inhibits the conversion of VSMCs from contractile phenotype into synthetic type via α2AR.

### DEX activates the GSK-3β/MKP-1/NRF2 pathway

**Fig. 5:**
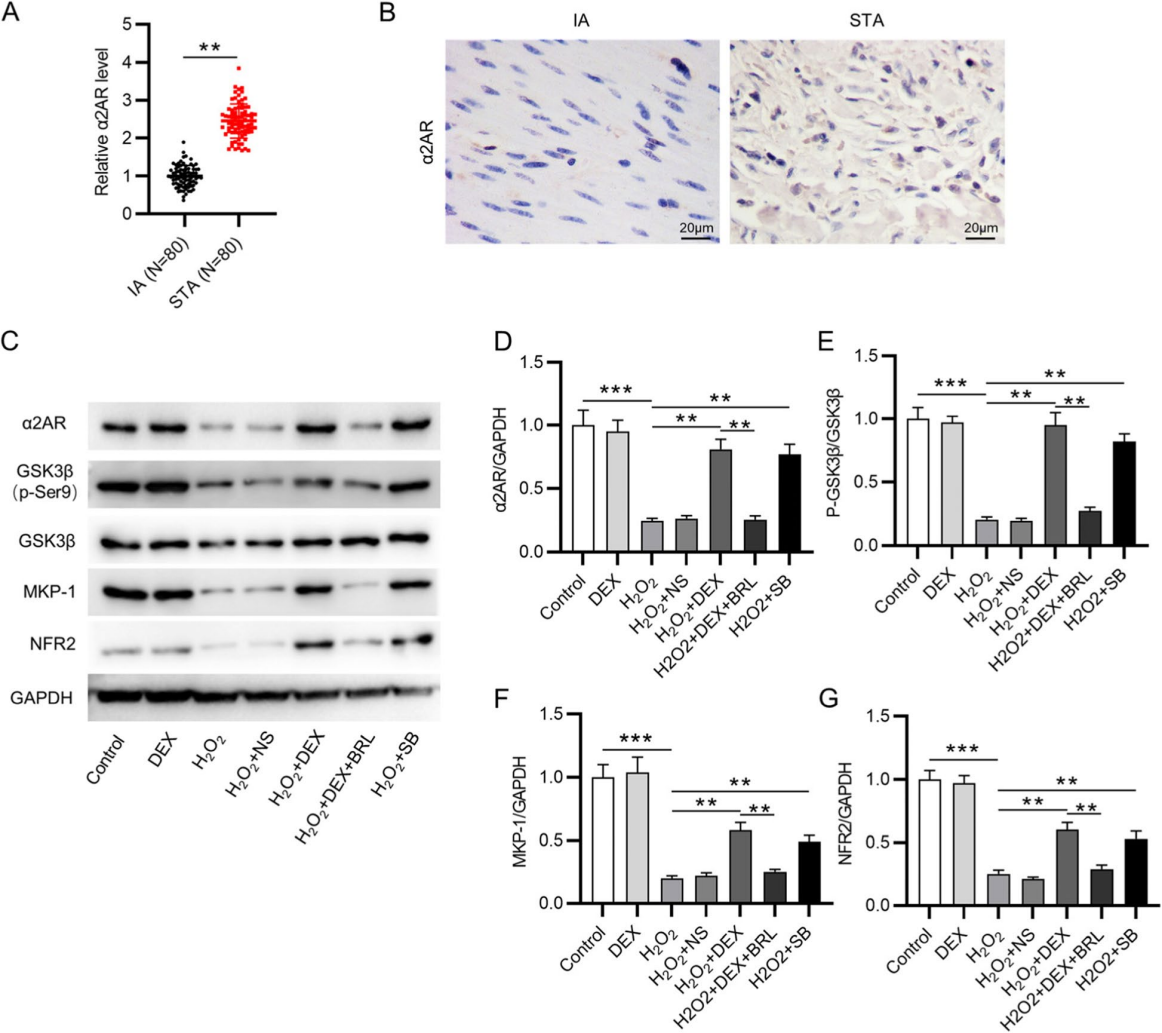
DEX regulates the GSK-3β/MKP-1/Nrf2 pathway. **A** RT-qPCR of α2AR level in IA and STA. (*N* = 80). **B** IHC of α2AR level in IA and STA. **C**-**G** The protein levels of α2AR, p-GSK-3β/ GSK-3β, MKP-1, and NRF2 were measured by western blotting. **p < 0.01, ***p < 0.001. DEX, dexmedetomidine; BRL, BRL44408, α2AR inhibitor; SB, SB216763, GSK-3β inhibitor

**Fig. 6:**
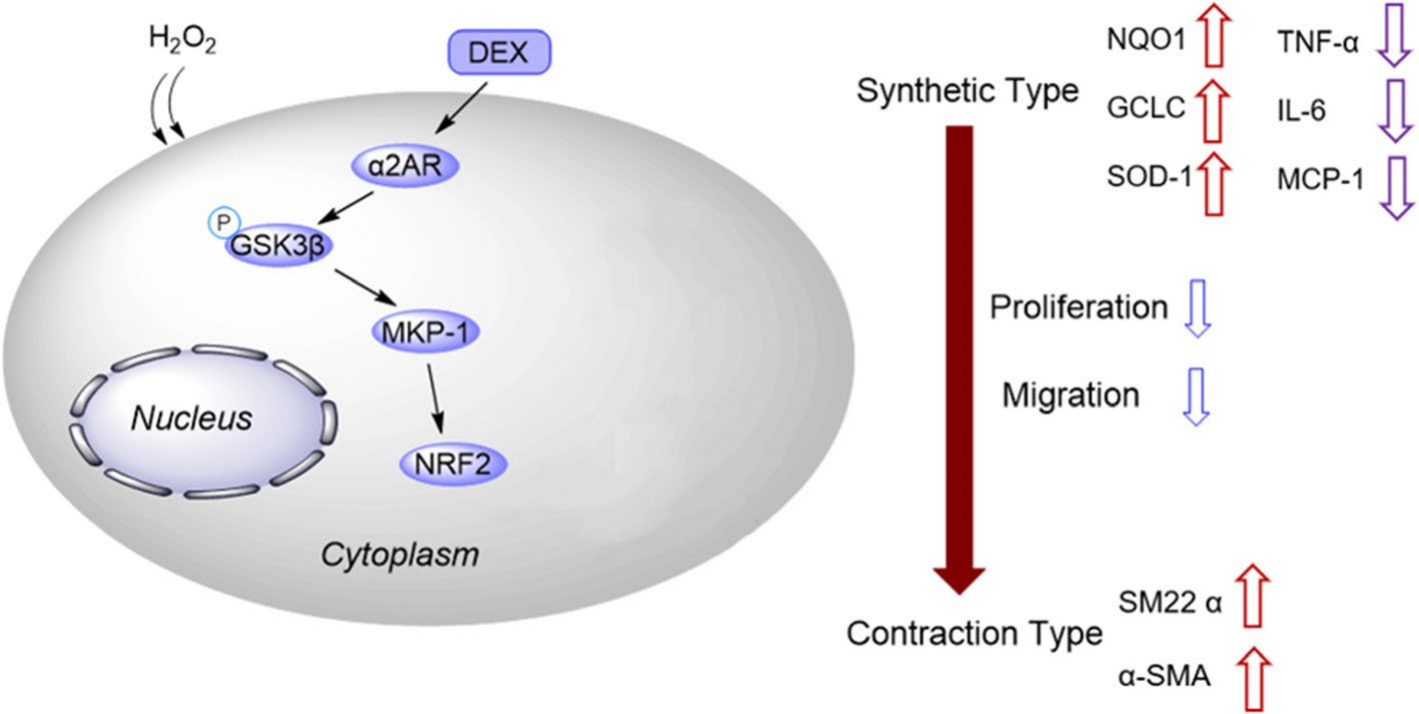
Schematic diagram of the mechanism of DEX against H_2_O_2_-induced cell malignancy

Compared with that in STA, the level of α2AR was downregulated in IAs, as shown by RT-qPCR (Fig. 5A). The results of immunohistochemistry staining demonstrated that α2AR expression was decreased in IAs compared with that in STAs (Fig. 5B). Finally, as western blotting showed, DEX or SB ameliorated the H2O2-induced inhibition in the protein levels of α2AR, phosphorylated GSK-3β, MKP-1, and NRF2, which were reduced by BRL (Fig. 5C-G). Figure 6 presented the schematic diagram showing the mechanism of DEX against H_2_O_2_-induced VSMC injury. DEX promoted the activation of GSK-3β/MKP-1/NRF2 pathway via the α2AR to increase the SM22α and αSMA levels and inhibit oxidative stress, leading to the conversion of VSMCs from synthetic type into contractile phenotype, thereby suppressing the proliferation, migration, and invasion of VSMCs.

## Discussion

The rupture of IA leads to SAH, causing severe mortality and morbidity [37]. Inflammation, phenotypic shift of smooth muscle cells, and ECM remodeling are three major contributors to IA formation and progression [38]. Herein, we detected the effects of DEX and its related mechanism in IA formation and progression.

As a highly selective α2AR agonist, DEX works in the intensive care unit as an anesthetic adjunct for patient sedation and in the operating room settings for general anesthesia [39]. Accumulating studies have confirmed the protective effects of DEX. For example, in IA treatment, DEX application ensures the stability of arterial pressure and reduces the injury to the postoperative brain functions caused by intraoperative hypoxia and brain ischemia [40]. During the first 24 h after admission, low dosage DEX leads to favorable neurological outcomes in patients with SAH [41]. DEX alleviates the lipopolysaccharide-induced inflammation responses to microglia cells by suppressing glycolysis [42]. DEX alleviates hippocampal inflammation to protect aged rats against postoperative cognitive dysfunction [43]. DEX ameliorates the oxygen–glucose deprivation/reperfusion-induced oxidative damage and neuronal apoptosis [44]. DEX provides neuroprotection via inhibition of oxidative damage following traumatic brain injury [45]. We herein first found that DEX moderated the inhibitory effects of H_2_O_2_ on cell viability and restored the promotion in cell migration and invasion. SM22 α and αSMA are the markers of contractile phenotype. Herein, DEX reversed the inhibitory effects of H_2_O_2_ on SM22 α and αSMA levels. NQO-1 and SOD1 are antioxidant enzymes, and TNF-α, IL-6 and MCP-1 participate in inflammation initiation [46]. GCLC limits the rate of GSH synthesis, and GSH protects against oxidative stress [47, 48]. In the current study, the reduction in the levels of NQO-1 and SOD1 as well as the upregulation in GCLC level induced by DEX indicated that DEX alleviated the oxidative damage. The restoration of increased TNF-α, IL-6 and MCP-1 levels following DEX demonstrated that DEX ameliorated inflammation responses. Therefore, we drawn a conclusion that DEX treatment led to the conversion of VSMCs from synthetic type into contractile phenotype.

DEX is reported to exert neuroprotective efficacy in patients with IA [40]. The underlying mechanism of DEX is complicated. DEX reduces autophagy and inflammation in rats with ischemia/reperfusion injury through the JNK signaling pathway [49]. In rats with traumatic brain injury, DEX alleviates the early neuronal damage via suppression of inflammation through the TLR4/NF-κB pathway [50]. DEX reverses the hypoxia/reoxygenation injury-induced oxidative stress by regulating the SIPT1/CHOP signaling pathway [51]. In a previous study, the authors found that DEX treatment results in the inactivation of GSK-3β via α2AR, thereby activating the MKP-1/NRF2 pathway to improve antioxidant capacity [52]. We herein discovered that DEX increased the protein levels of phosphorylated GSK-3β, MKP-1, NRF2 and α2AR, suggesting that DEX regulated the GSK-3β/MKP-1/NRF2 pathway. To further verify that the functions of DEX were mediated by GSK-3β, SB216763 was used to inhibit GSK-3β, and we found that SB216763 treatment restored the H2O2-induced promotion in ROS generation, cell migration, cell invasion, inflammatory responses, and oxidative stress.

In summary, this study demonstrated the protective roles of DEX against H2O2-induced cell proliferation, invasion, migration, oxidative damage, and inflammation responses, leading to the conversion of VSMCs from synthetic type to contraction type by via the α2AR/GSK-3β/MKP-1/NRF2 axis. To be honest, there are some limitations in this study. First, in vivo experiments and more comprehensive studies are needed in the subsequent studies. Second, the studies on α2AR are inadequate. Despite these limitations, we believed that our study would provide a theoretical basis for IA treatment.

## Supplementary Information

**Additional file 1.**

## Data Availability

The datasets used during the current study are available from the corresponding author on reasonable request. The data are not publicly available due to privacy or ethical restrictions.

## References

[1] Juvela S, Poussa K, Porras M (2001). Factors affecting formation and growth of intracranial aneurysms: a long-term follow-up study. Stroke.

[2] Brisman JL, Song JK, Newell DW (2006). Cerebral aneurysms. N Engl J Med.

[3] Korja M, Kivisaari R, Rezai Jahromi B, Lehto H (2017). Size and location of ruptured intracranial aneurysms: consecutive series of 1993 hospital-admitted patients. J Neurosurg.

[4] Jiang Z, Huang J, You L, Zhang J, Li B (2021). Pharmacological inhibition of STAT3 by BP-1-102 inhibits intracranial aneurysm formation and rupture in mice through modulating inflammatory response. Pharmacol Res Perspect.

[5] Kleinloog R, de Mul N, Verweij BH, Post JA, Rinkel GJE, Ruigrok YM (2018). Risk Factors for intracranial aneurysm rupture: a systematic review. Neurosurgery.

[6] Signorelli F, Sela S, Gesualdo L, Chevrel S, Tollet F, Pailler-Mattei C (2018). Hemodynamic stress, inflammation, and intracranial aneurysm development and rupture: a systematic review. World neurosurgery.

[7] Chalouhi N, Hoh BL, Hasan D (2013). Review of cerebral aneurysm formation, growth, and rupture. Stroke.

[8] Liu P, Song Y, Zhou Y, Liu Y, Qiu T, An Q (2018). Cyclic Mechanical stretch induced smooth muscle cell changes in cerebral aneurysm progress by reducing collagen Type IV and collagen type VI levels. Cell Physiol Biochem.

[9] Lacolley P, Regnault V, Nicoletti A, Li Z, Michel JB (2012). The vascular smooth muscle cell in arterial pathology: a cell that can take on multiple roles. Cardiovasc Res.

[10] Owens GK, Kumar MS, Wamhoff BR (2004). Molecular regulation of vascular smooth muscle cell differentiation in development and disease. Physiol Rev.

[11] Starke RM, Chalouhi N, Ding D, Raper DM, McKisic MS, Owens GK (2014). Vascular smooth muscle cells in cerebral aneurysm pathogenesis. Transl Stroke Res.

[12] Wang G, Jacquet L, Karamariti E, Xu Q (2015). Origin and differentiation of vascular smooth muscle cells. J Physiol.

[13] Chalouhi N, Ali MS, Jabbour PM, Tjoumakaris SI, Gonzalez LF, Rosenwasser RH (2012). Biology of intracranial aneurysms: role of inflammation. J Cereb Blood Flow Metab.

[14] Javahertalab M, Susanabadi A, Modir H, Kamali A, Amani A, Almasi-Hashiani A (2020). Comparing intravenous dexmedetomidine and clonidine in hemodynamic changes and block following spinal anesthesia with ropivacaine in lower limb orthopedic surgery: a randomized clinical trial. Med Gas Res.

[15] Silpa AR, Koshy KA, Subramanian A, Pradeep KK (2020). Comparison of the efficacy of two doses of dexmedetomidine in attenuating the hemodynamic response to intubation in patients undergoing elective cardiac surgery: a randomized double-blinded study. J Anaesthesiol Clin Pharmacol.

[16] Tang C, Huang X, Kang F, Chai X, Wang S, Yin G (2015). Intranasal dexmedetomidine on stress hormones, inflammatory markers, and postoperative analgesia after functional endoscopic sinus surgery. Mediators Inflamm.

[17] Prontera A, Baroni S, Marudi A, Valzania F, Feletti A, Benuzzi F (2017). Awake craniotomy anesthetic management using dexmedetomidine, propofol, and remifentanil. Drug Des Dev Ther.

[18] Kim JK (2016). An introduction to the various role of dexmedetomidine. Korean J Anesthesiol.

[19] Zhao P, Zhou R, Zhu XY, Hao YJ, Li N, Wang J (2015). Matrine attenuates focal cerebral ischemic injury by improving antioxidant activity and inhibiting apoptosis in mice. Int J Mol Med.

[20] Bilgi KV, Vasudevan A, Bidkar PU (2016). Comparison of dexmedetomidine with fentanyl for maintenance of intraoperative hemodynamics in hypertensive patients undergoing major surgery: a randomized controlled trial. Anesth Essays Res.

[21] Wang Y, Han R, Zuo Z (2016). Dexmedetomidine post-treatment induces neuroprotection via activation of extracellular signal-regulated kinase in rats with subarachnoid haemorrhage. Br J Anaesth.

[22] Sriganesh K, Reddy M, Jena S, Mittal M, Umamaheswara Rao GS (2015). A comparative study of dexmedetomidine and propofol as sole sedative agents for patients with aneurysmal subarachnoid hemorrhage undergoing diagnostic cerebral angiography. J Anesth.

[23] Erdman MJ, Doepker BA, Gerlach AT, Phillips GS, Elijovich L, Jones GM (2014). A comparison of severe hemodynamic disturbances between dexmedetomidine and propofol for sedation in neurocritical care patients. Crit Care Med.

[24] Farr SA, Ripley JL, Sultana R, Zhang Z, Niehoff ML, Platt TL (2014). Antisense oligonucleotide against GSK-3β in brain of SAMP8 mice improves learning and memory and decreases oxidative stress: Involvement of transcription factor Nrf2 and implications for Alzheimer disease. Free Radical Biol Med.

[25] Talwar H, Bauerfeld C, Bouhamdan M, Farshi P, Liu Y, Samavati L (2017). MKP-1 negatively regulates LPS-mediated IL-1β production through p38 activation and HIF-1α expression. Cell Signal.

[26] Force T, Woodgett JR (2009). Unique and overlapping functions of GSK-3 isoforms in cell differentiation and proliferation and cardiovascular development. J Biol Chem.

[27] Jiang L, Xia QJ, Dong XJ, Hu Y, Chen ZW, Chen K (2017). Neuroprotective effect of breviscapine on traumatic brain injury in rats associated with the inhibition of GSK3β signaling pathway. Brain Res.

[28] Zheng Y, Im CN, Seo JS (2006). Inhibitory effect of Hsp70 on angiotensin II-induced vascular smooth muscle cell hypertrophy. Exp Mol Med.

[29] Hu Y, Li H, Li R, Wu Z, Yang W, Qu W (2020). Puerarin protects vascular smooth muscle cells from oxidized low-density lipoprotein-induced reductions in viability via inhibition of the p38 MAPK and JNK signaling pathways. Exp Ther Med.

[30] Scannevin RH, Chollate S, Jung MY, Shackett M, Patel H, Bista P (2012). Fumarates promote cytoprotection of central nervous system cells against oxidative stress via the nuclear factor (erythroid-derived 2)-like 2 pathway. J Pharmacol Exp Ther.

[31] Meng YY, Wu CW, Yu B, Li H, Chen M, Qi GX (2018). PARP-1 Involvement in autophagy and their roles in apoptosis of vascular smooth muscle cells under oxidative stress. Folia Biol.

[32] Zhao W, Zhang H, Su JY (2018). MicroRNA-29a contributes to intracranial aneurysm by regulating the mitochondrial apoptotic pathway. Mol Med Rep.

[33] Ding X, Wang X, Han L, Zhao Z, Jia S, Tuo Y (2021). CircRNA DOCK1 Regulates miR-409-3p/MCL1 axis to modulate proliferation and apoptosis of human brain vascular smooth muscle cells. Front Cell Dev Biol.

[34] Huang J, Zhang H, You L, Zhang J, Jiang Z (2022). Coenzyme Q10 inhibits intracranial aneurysm formation and progression in a mouse model. Pediatr Res.

[35] Movahedan A, Majdi M, Afsharkhamseh N, Sagha HM, Saadat NS, Shalileh K (2012). Notch inhibition during corneal epithelial wound healing promotes migration. Invest Ophthalmol Vis Sci.

[36] Tian H, Hou L, Xiong Y, Cheng Q, Huang J (2019). Effect of dexmedetomidine-mediated insulin-like growth factor 2 (IGF2) signal pathway on immune function and invasion and migration of cancer cells in rats with ovarian cancer. Med Sci Monit.

[37] Thompson BG, Brown RD, Amin-Hanjani S, Broderick JP, Cockroft KM, Connolly ES (2015). Guidelines for the management of patients with unruptured intracranial aneurysms: a Guideline for healthcare professionals from the american heart association/american stroke association. Stroke.

[38] Keedy A (2006). An overview of intracranial aneurysms. McGill J Med.

[39] Gao J, Sun Z, Xiao Z, Du Q, Niu X, Wang G (2019). Dexmedetomidine modulates neuroinflammation and improves outcome via alpha2-adrenergic receptor signaling after rat spinal cord injury. Br J Anaesth.

[40] Zheng D, Zhao S, Zhang N, Shi J (2020). Brain protective effect and hemodynamics of dexmedetomidine hydrochloride in patients with intracranial aneurysm. Saudi J Biol Sci.

[41] Okazaki T, Hifumi T, Kawakita K, Shishido H, Ogawa D, Okauchi M (2018). Association between dexmedetomidine use and neurological outcomes in aneurysmal subarachnoid hemorrhage patients: a retrospective observational study. J Crit Care.

[42] Meng F, Yu W, Duan W, Wang T, Liu Y (2020). Dexmedetomidine attenuates LPS-mediated BV2 microglia cells inflammation via inhibition of glycolysis. Fundam Clin Pharmacol.

[43] Chen N, Chen X, Xie J, Wu C, Qian J (2019). Dexmedetomidine protects aged rats from postoperative cognitive dysfunction by alleviating hippocampal inflammation. Mol Med Rep.

[44] Xu D, Zhou C, Lin J, Cai W, Lin W (2021). Dexmedetomidine provides protection to neurons against OGD/R-induced oxidative stress and neuronal apoptosis. Toxicol Mech Methods.

[45] Li F, Wang X, Deng Z, Zhang X, Gao P, Liu H (2018). Dexmedetomidine reduces oxidative stress and provides neuroprotection in a model of traumatic brain injury via the PGC-1α signaling pathway. Neuropeptides.

[46] Qiu Y, Yang J, Wang L, Yang X, Gao K, Zhu C (2021). Dietary resveratrol attenuation of intestinal inflammation and oxidative damage is linked to the alteration of gut microbiota and butyrate in piglets challenged with deoxynivalenol. J Anim Sci Biotechnol.

[47] Lu SC (2013). Glutathione synthesis. Biochem Biophys Acta.

[48] Espinosa-Díez C, Miguel V, Vallejo S, Sánchez FJ, Sandoval E, Blanco E (2018). Role of glutathione biosynthesis in endothelial dysfunction and fibrosis. Redox Biol.

[49] Zhu Y, Li S, Liu J, Wen Q, Yu J, Yu L (2019). Role of JNK signaling pathway in dexmedetomidine post-conditioning-induced reduction of the inflammatory response and autophagy effect of focal cerebral ischemia reperfusion injury in rats. Inflammation.

[50] Huang GR, Hao FG (2021). Dexmedetomidine inhibits inflammation to alleviate early neuronal injury via TLR4/NF-κB pathway in rats with traumatic brain injury. Crit Rev Eukaryot Gene Expr.

[51] Zhang Y, Zhao Q, Li X, Ji F (2021). Dexmedetomidine reversed hypoxia/reoxygenation injury-induced oxidative stress and endoplasmic reticulum stress-dependent apoptosis of cardiomyocytes via SIRT1/CHOP signaling pathway. Mol Cell Biochem.

[52] Sha J, Zhang H, Zhao Y, Feng X, Hu X, Wang C (2019). Dexmedetomidine attenuates lipopolysaccharide-induced liver oxidative stress and cell apoptosis in rats by increasing GSK-3β/MKP-1/Nrf2 pathway activity via the α2 adrenergic receptor. Toxicol Appl Pharmacol.

