# Publisher Correction to: Dexmedetomidine alleviates inflammatory response and oxidative stress injury of vascular smooth muscle cell via α2AR/GSK-3β/MKP-1/NRF2 axis in intracranial aneurysm

**DOI:** 10.1186/s40360-022-00639-6

**Published:** 2023-01-30

**Authors:** Ze Zhang, Xiue Mu, Xiaohui Zhou

**Affiliations:** Department of Anesthesiology, The First Hospital of Hebei Medical University, 89 Donggang Road, Shijiazhuang, 050000 Hebei China

**Correction: BMC Pharmacol Toxicol 23, 81 (2022)**

**https://doi.org/10.1186/s40360-022-00607-0**

During the publication process of the original article [1] there was an error in updating Fig. 3b and c. The correct and incorrect figure are shown in this correction article. The original publication has been updated. The publisher apologizes for the inconvenience caused to the authors & readers.

**Figure 1** Incorrect figure 3 as originally published

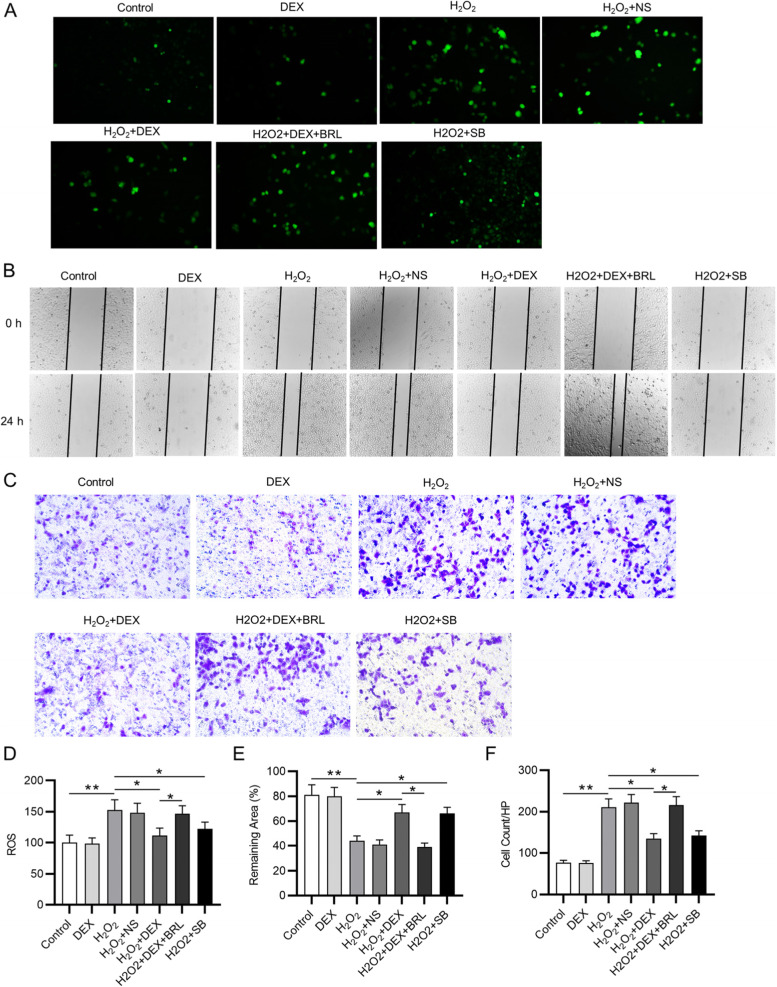



**Figure 2** correct version of figure 3


