# Supplementary material for: Dexmedetomidine alleviates inflammatory response and oxidative stress injury of vascular smooth muscle cell via α2AR/GSK-3β/MKP-1/NRF2 axis in intracranial aneurysm
Source: BMC Pharmacol Toxicol. 2022 Oct 23;23:81. doi: 10.1186/s40360-022-00607-0 (PMC9588221; doi:10.1186/s40360-022-00607-0)

Supplementary Information: Uncropped blot images.

Figure 4A

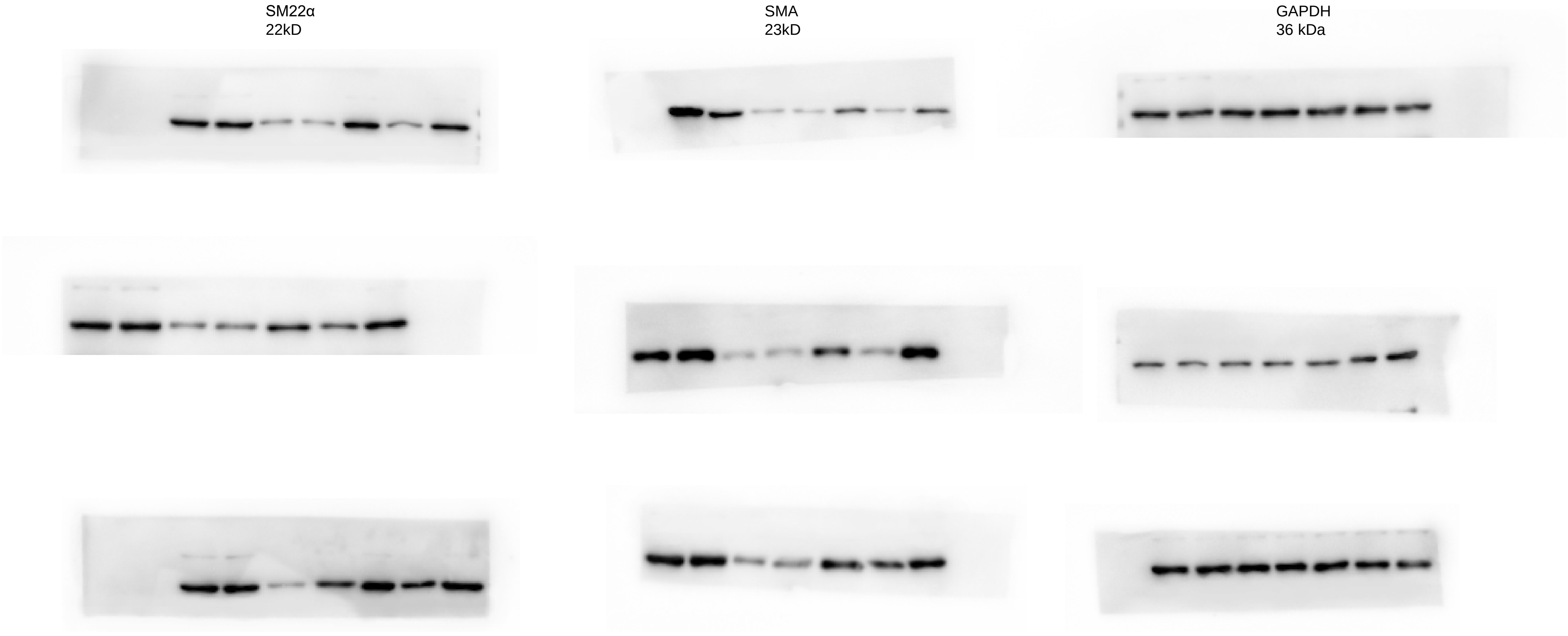

Figure 5C

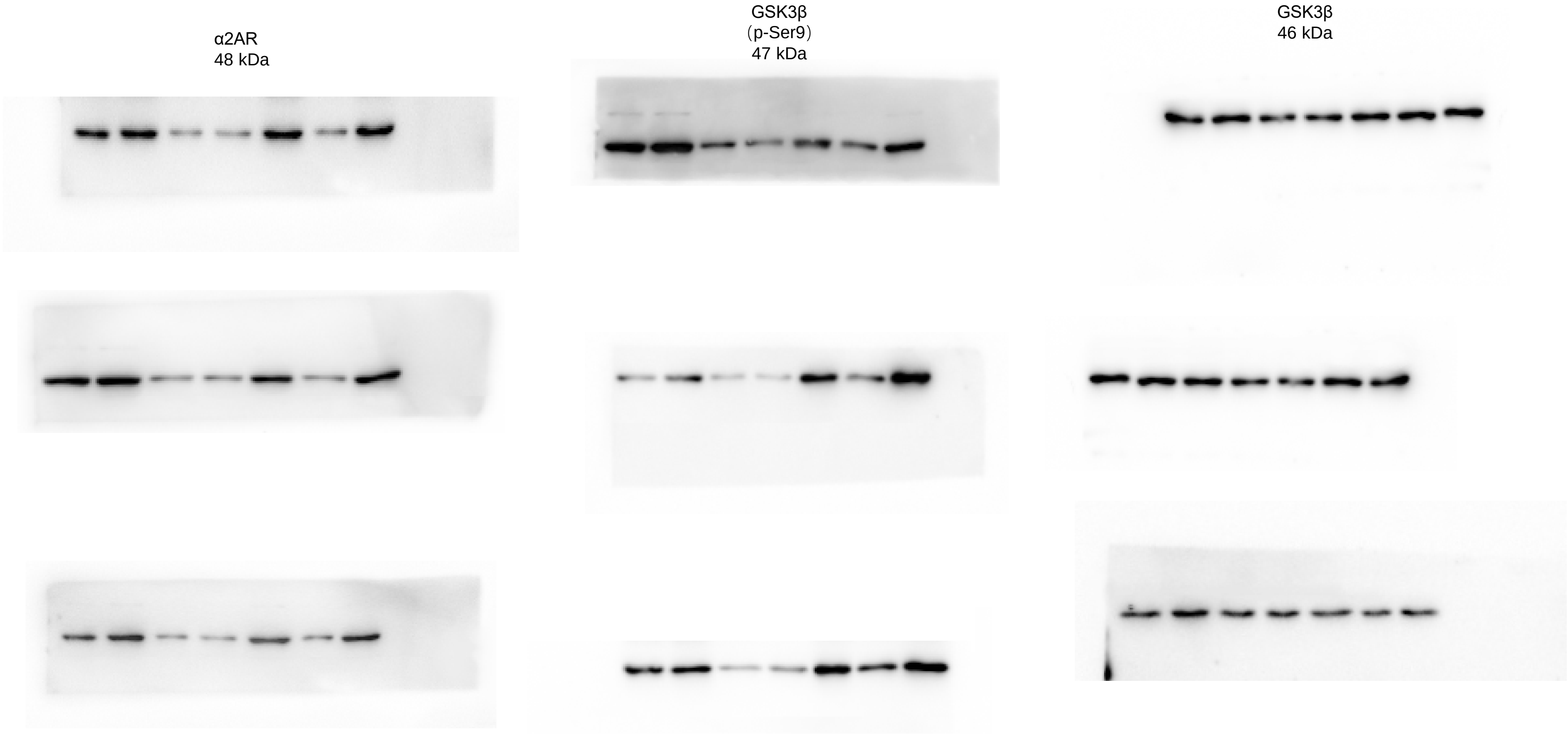

Figure 5C

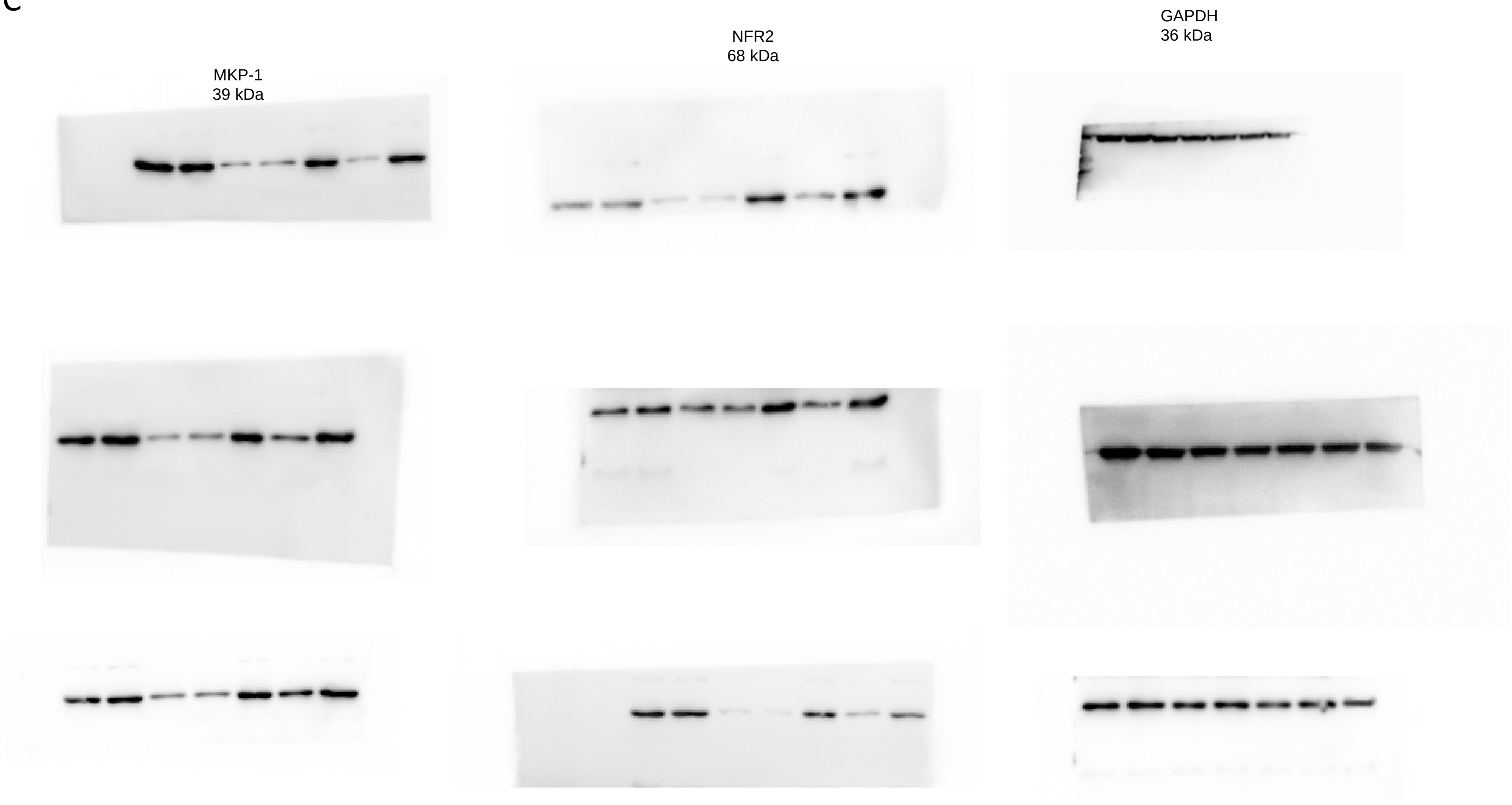

Supplement: Supplementary file 1 — Additional file 1. [file 40360_2022_607_MOESM1_ESM.pdf]
